# Rational Design of a Surface Acoustic Wave Device for Wearable Body Temperature Monitoring

**DOI:** 10.3390/mi15050555

**Published:** 2024-04-23

**Authors:** Yudi Xie, Minglong Deng, Jinkai Chen, Yue Duan, Jikai Zhang, Danyu Mu, Shurong Dong, Jikui Luo, Hao Jin, Shoji Kakio

**Affiliations:** 1 Ministry of Education Key Laboratory of RF Circuits and Systems, Hangzhou Dianzi University, Hangzhou 310018, China; 2 College of Information Science and Electronic Engineering, Zhejiang University, Hangzhou 310027, China; 3 International Joint Innovation Center, Zhejiang University, Haining 314400, China; 4 Integrated Graduate School of Medicine, Engineering, and Agricultural Sciences, University of Yamanashi, Kofu 400-8511, Japan; kakio@yamanashi.ac.jp

**Keywords:** passive wireless sensing, surface acoustic wave, temperature sensor, COM model

## Abstract

Continuous monitoring of vital signs based on advanced sensing technologies has attracted extensive attention due to the ravages of COVID-19. A maintenance-free and low-cost passive wireless sensing system based on surface acoustic wave (SAW) device can be used to continuously monitor temperature. However, the current SAW-based passive sensing system is mostly designed at a low frequency around 433 MHz, which leads to the relatively large size of SAW devices and antenna, hindering their application in wearable devices. In this paper, SAW devices with a resonant frequency distributed in the 870 MHz to 960 MHz range are rationally designed and fabricated. Based on the finite-element method (FEM) and coupling-of-modes (COM) model, the device parameters, including interdigital transducer (IDT) pairs, aperture size, and reflector pairs, are systematically optimized, and the theoretical and experimental results show high consistency. Finally, SAW temperature sensors with a quality factor greater than 2200 are obtained for real-time temperature monitoring ranging from 20 to 50 °C. Benefitting from the higher operating frequency, the size of the sensing system can be reduced for human body temperature monitoring, showing its potential to be used as a wearable monitoring device in the future.

## 1. Introduction

In recent years, advanced sensing technologies have been in a stage of rapid development as the core components of the perception layer of the new generation of information technology industries such as the Internet of Things, artificial intelligence, etc. [1,2,3]. Meanwhile, due to the ravages of COVID-19, continuous monitoring of vital signs based on advanced sensing technologies has attracted more and more attention [4,5], as the long-term monitoring of body temperature, heart rate, respiration, and other parameters is helpful to infer whether the human body is healthy or not, which plays an increasingly important role in the diagnosis and follow-up treatment of the human body [6,7]. Among the vital signs, human body temperature is one of the most important and urgent parameters that should be monitoring continuously.

Long-term temperature monitoring in existing wearable devices can be achieved by wired or wireless methods. The wired temperature monitoring method introduces the cable into the system, which will lead to inconvenience in daily life. Therefore, long-term wireless temperature monitoring using a wireless method is preferred. A traditional active wireless sensing system can achieve temperature monitoring. However, such a method has a great demand for batteries at the sensing end, as all the components including sensors and the wireless transmission module consume a large amount of energy [8]. For example, Alam et al. proposed a wearable multi-point body temperature and sweat analysis device. The device communicates wirelessly through Bluetooth and can work continuously for ten hours [9]. YUN et al. proposed a body temperature paste based on Bluetooth, which can detect body temperature in real time, but its own flexible battery can only provide about 38 h of endurance [10]. In summary, based on the traditional active wireless temperature monitoring system, it is hard to achieve properties including the comfort of wearing, miniaturized system size, low cost, and long operation time at the same time.

Compared to the traditional active wireless sensing system, a passive wireless sensing system can also be used to continuously monitor temperature with nearly zero energy consumption at the sensing end. Typical passive wireless sensing systems are usually based on inductor/capacitor (LC) and surface acoustic wave (SAW) devices. Wang et al. proposed an LC passive wireless sensor for temperature monitoring, which has a sensitivity of 4.11 MHz/°C in the range of 36–40 °C and a resolution of 0.1 °C, but the transmission distance is only several centimeters due to the limitation of the coil coupling [11]. Varadharajan et al. proposed an LC resonator capable of operating in the range of 200–1200 °C with a temperature sensitivity of 170 kHz/°C for harsh environments, but its optimal operating distance from the reader is only 2.5 cm [12]. It is clear that an LC passive wireless sensing system can only achieve near-field wireless signal transmission due to the huge energy loss in inductive/capacitive coupling at long range. Compared to the LC passive wireless sensing system, an SAW-based passive wireless sensing system can achieve much longer transmission distance due to the higher quality factor of SAW devices [13,14]. Gao et al. proposed a SAW temperature sensor with an operating frequency of about 430 MHz and a detection range of −30–100 °C, which can achieve wireless transmission of 0.5 m [15]. However, the current SAW-based passive wireless sensors are mostly designed at a low resonant frequency around 433 MHz, which leads to the relatively large size of SAW devices and antenna, hindering their application in wearable devices.

In this paper, SAW temperature sensors with a resonant frequency distributed in the 870 MHz to 960 MHz range are proposed for wearable body temperature monitoring. The sensors are rationally designed based on a coupling-of-modes (COM) model. The parameters of the COM model are obtained by the finite-element method (FEM), and the device parameters, including interdigital transducer (IDT) pairs, aperture size, and reflector pairs, are systematically optimized, and the theoretical and experimental results show high consistency. Finally, SAW temperature sensors with a quality factor greater than 2200 are obtained. Based on a previously developed wireless sensing system, real-time body temperature monitoring can be achieved, showing its potential to be used in wearable monitoring devices in the human body.

## 2. Rationally Theoretical Design of SAW Devices

### 2.1. Design Process

**Figure 1:**
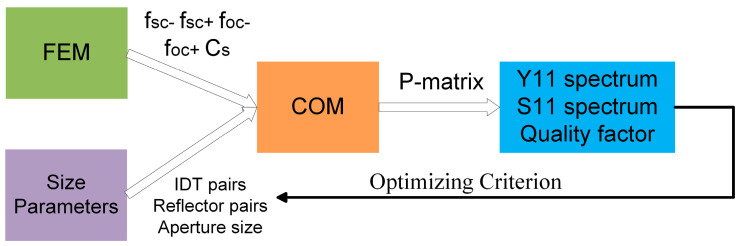
Design process of SAW devices.

The design process of SAW devices is shown in Figure 1. Firstly, FEM simulation is conducted through COMSOL to obtain basic parameters including short-circuited grating stopband frequencies (*f_sc__−_* and *f_sc__+_*), open-circuited grating stopband frequencies (*f_oc__−_* and *f_oc__+_*), and the unit static capacitance *C_s_*. Then, these parameters are brought into the COM model for calculation of the Y11/S11 spectrum and optimization. The optimizing criteria include size limitations and quality factor (Q factor) limitations. Based on the optimizing criteria, the geometric size parameters of SAW devices such as IDT pairs, reflector pairs, and aperture size *W* can be obtained for subsequent device fabrication.

### 2.2. COM Parameters Extraction Using FEM

As the COM model is a phenomenological model [16], the extracted parameters (*f_sc__−_*, *f_sc__+_*, *f_oc__−_*, *f_oc__+_*, and *C_s_*) should be as accurate as possible to ensure the reliable simulation results of SAW devices. FEM (i.e., COMSOL Multiphysics) is the most commonly used for COM parameters’ extraction [17,18,19,20].

**Figure 2:**
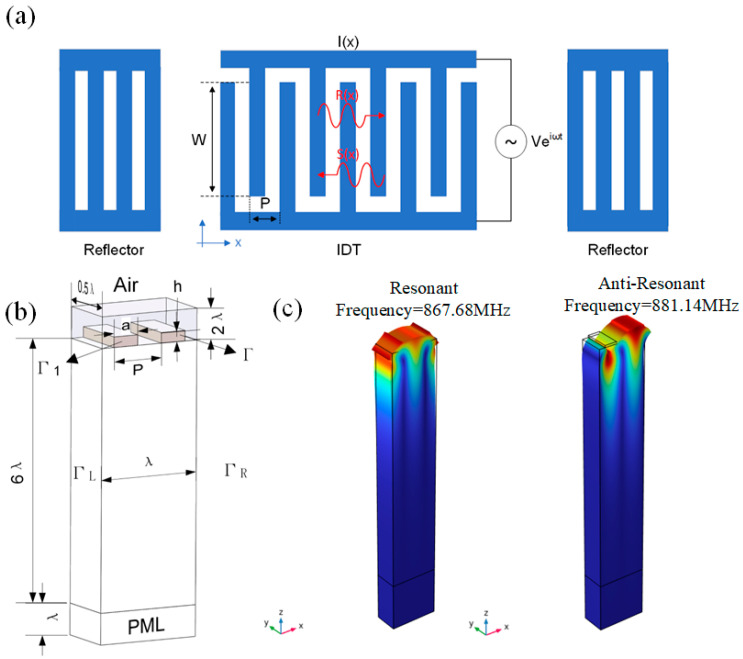
Schematic diagram of (**a**) a one-port SAW resonator and (**b**) 3D geometric FEM model of the SAW device. (**c**) Simulated vibration shape of the SAW device with a 867.68 MHz (*f_sc−_*) and 881.14 MHz (*f_sc+_*) stopband frequency.

In order to minimize the size of the SAW device and antenna for wearable applications, the operating frequency of the device is designed to be distributed within the range of 870 MHz to 960 MHz. The frequency band was divided into 15 frequencies for future multi-location temperature monitoring. Uniform aluminium IDT was chosen as shown in Figure 2a. The IDT metallization ratio *η* was set to be 0.5. YX quartz was chosen as the piezoelectric substrate, resulting in a phase velocity *v* of 3159 m/s [21]. According to the following equation, the wavelength *λ* and the correspondence IDT width can be roughly determined for the FEM model,

(1) $$\lambda =\frac{\nu }{f}$$

In practice, 15 operating frequencies were determined in the range of 870 MHz to 960 MHz with 3.216 µm as the starting wavelength value and 0.024 µm as the incremental wavelength step size. Subsequently, the size parameters were brought into the periodical FEM model as shown in Figure 2b. The length, width, and height of the 3D FEM model were *λ*, 0.5*λ*, and 6*λ*, respectively. Considering the influence of air, a layer of air with a height of 2*λ* was added above the quartz material. For the boundary conditions, both Γ_1_ and Γ_2_ were set to be grounded or floating potentials corresponds to the short-circuit and open-circuit condition, respectively. The periodic boundary condition was set to Γ_L_ and Γ_R_. Lastly, a perfectly matched layer (PML) with a height of *λ* was added under the piezoelectric substrate. After that, the FEM model could be simulated to obtain the *f_sc__−_*, *f_sc__+_*, *f_oc__−_*, and *f_oc__+_*, and the vibration shape of the SAW device with a 867.68 MHz (*f_sc__−_*) and 881.14 MHz (*f_sc__+_*) stopband frequency, which was designed to operate at 870MHz, is demonstrated in Figure 2c.

In addition, through the steady-state simulation, the unit static capacitance *C_s_* could also be calculated. Finally, based on the extracted data, the essential COM model parameters, including center frequency *f*, acoustic velocity *v*, reflection coefficient *κ*, and conversion coefficient *α_n_*, could be calculated using the combination of Equation (1) and the following equation sets,

(2) $$\left\{\begin{array}{c} f=\frac{\left(f_{sc+}\;+\;f_{sc-}\right)}{2}\\ \left|\kappa \right|\lambda =2\pi \frac{f_{sc+}\;-\;f_{sc-}}{f_{sc+}\;+\;f_{sc-}}\\ |\alpha_{n}|=\sqrt{\frac{\omega C_{s}W\pi }{\lambda^{2}}(\frac{f_{oc+}\;+\;f_{oc-}}{f_{sc+}\;+\;f_{sc-}}-1)} \end{array}\right.$$

### 2.3. SAW Device Performance Calculation Using COM Model

The COM model was first proposed by Pierce in 1954 [22]. Cross et al. first applied it to the simulation and design analysis of SAW devices in 1976 [23,24]. Since then, the COM model has been continuously developed [25,26,27,28,29]. With the improvement of Chen et al., the second-order effects such as surface acoustic wave reflection, wave velocity change, and loss attenuation are taken into account, so that it can simulate SAW devices more accurately and effectively [30,31,32,33,34].

As shown in Figure 2a, a one-port SAW resonator consisting of one IDT with reflective gratings on either side was rationally designed. In the COM model, the AC driving voltage V loaded on the bus bar excites acoustic waves on the surface of the piezoelectric material due to the inverse piezoelectric effect, so that there are two acoustic waves propagating in the opposite direction. One propagates in the positive direction of the X-axis and the other propagates in the negative direction of the X-axis, which are represented by *R(x)* and *S(x)*. At the same time, the acoustic wave propagating on the surface of the piezoelectric material generates a current on the IDT due to the piezoelectric effect, which is expressed by *I(x)*.

*R(x)* and *S(x)* are coupled with each other through multiple reflections in the metal gate. After introducing attenuation, the governing equation of COM can be obtained as follows:

(3) $$\left\{\begin{array}{c} \frac{dR_{0}}{dx}=j\kappa S_{0}\exp(2j\Delta x)+j\alpha V\exp(j\Delta x)\\ \frac{dS_{0}}{dx}=-j\kappa^{*}R_{0}\exp(-2j\Delta x)-j\alpha^{*}V\exp(-j\Delta x)\\ \frac{dI}{dx}=-2j\alpha^{*}R_{0}\exp(-j\Delta x)-2j\alpha S_{0}\exp(j\Delta x)+j\omega C_{s}V \end{array}\right.$$

where Δ is the detuning parameter:

(4) $$\Delta =k-k_{0}=k_{r}\;-\;k_{0}\;-\;j\gamma =\frac{\omega }{v}-k_{0}-j\gamma$$

*k* is the complex wave number, the real part *k_r_* represents the average propagation constant when multiple reflections are ignored under the frequency *ω*, the imaginary part *γ* represents the attenuation, and *k*_0_ is the acoustic synchronous wave number of IDT. *v_e_*, *κ*, *α*, *C_s_*, and the attenuation coefficient *γ* are all independent parameters. When the resonator is a uniform structure, *κ* and *α* are real values and can be regarded as lossless propagation in the Rayleigh wave mode, that is, the attenuation coefficient *γ* = 0.

In the case of a short-circuit grating, the metal electrodes are connected to each other through the bus, so the induced potential V generated by the surface acoustic wave is zero. The homogeneous form of Equation (3) is obtained:

(5) $$\left\{\begin{array}{c} \frac{dR_{0}}{dx}=j\kappa S_{0}\exp\left(2j\Delta x\right)\\ \frac{dS_{0}}{dx}=-j\kappa^{*}R_{0}\exp\left(-2j\Delta x\right) \end{array}\right.$$

The general solution of Equation (5) is as follows:

(6) $$\left\{\begin{array}{c} R_{0}=A_{+}\exp[j(\Delta +D)x]+A_{-}\exp[j(\Delta -D)x]\\ S_{0}=\frac{1}{\kappa }\{A_{+}(\Delta +D)\exp[-j(\Delta -D)x]+A_{-}(\Delta -D)\exp[-j(\Delta +D)x]\} \end{array}\right.$$

Among them, the values of undetermined coefficients A_+_ and A_−_ are determined by the boundary conditions, *D* is the dispersion relation of the COM equation, and the calculation method is as follows:

(7) $$D=\sqrt{\Delta^{2}-|\kappa {|}^{2}}$$

It can be seen from the above formula that when the five independent parameters (*κ*, *α*, *v_e_*, *C_s_*, *γ*) of the COM model are obtained, we can accurately obtain the propagation characteristics and stopband of the surface acoustic wave on the device.

According to the above calculation and analysis, we have obtained the propagation characteristics of a single IDT SAW device. In order to further obtain the working performance of the entire SAW device, we first need to use the P matrix to cascade the interdigital transducers. Assuming that the incident surface acoustic wave amplitudes of the left and right acoustic ends of the IDT are *a*_1_ and *a*_2_, respectively, the amplitudes generated by the IDT are *b*_1_ and *b*_2_, the voltage loaded on the IDT electrical end is V, and the current is *I*, the acoustic and electrical characteristics of the IDT can be described by (8):

(8) $$\left.\left(\begin{array}{c} b_{1}\\ b_{2}\\ I \end{array}\right.\right)=\left(\begin{array}{ccc} P_{11} & P_{12} & P_{13}\\ P_{21} & P_{22} & P_{23}\\ P_{31} & P_{32} & P_{33} \end{array}\right)\left(\begin{array}{c} a_{1}\\ a_{2}\\ V \end{array}\right)$$

wherein,

$$\left\{\begin{array}{c} P_{11}=\frac{j\kappa^{*}sin(DL)}{Dcos(DL)\;+\;j\Delta sin(DL)}\\ P_{12}=P_{21}=\frac{(-1{)}^{2N}D}{Dcos(DL)\;+\;j\Delta sin(DL)}\\ P_{13}=-\frac{1}{2}P_{31}=jL\frac{sin(DL/2)}{DL/2}\frac{j(\Delta \alpha^{*}\;+\;\kappa^{*}\alpha )sin(DL/2)\;+\;\alpha^{*}Dcos(DL/2)}{Dcos(DL)\;+\;j\Delta sin(DL)}\\ P_{22}=\frac{j\kappa sin(DL)}{Dcos(DL)\;+\;j\Delta sin(DL)}\\ P_{23}=-\frac{1}{2}P_{32}=(-1{)}^{2N}jL\frac{sin(DL/2)}{DL/2}\frac{j(\Delta \alpha \;+\;\kappa \alpha^{*})sin(DL/2)\;+\;\alpha Dcos(DL/2)}{Dcos(DL)\;+\;j\Delta sin(DL)}\\ P_{33}=-\frac{4}{D^{3}}\frac{\left[(\Delta^{2}\;+\;|\kappa {|}^{2})|\alpha {|}^{2}\;+\;2\Delta Re(\kappa^{*}\alpha^{2})][1\;-\;cos(DL)\right]}{Dcos(DL)\;+\;j\Delta sin(DL)}\\ +\;j\frac{4}{D^{2}}\frac{\left[\Delta |\alpha {|}^{2}\;+\;Re(\kappa^{*}\alpha^{2})]sin(DL\right)}{Dcos(DL)\;+\;j\Delta sin(DL)}\;-\;jL\frac{4}{\Delta^{2}\;-\;|\kappa {|}^{2}}[\Delta |\alpha {|}^{2}\;+\;Re(\kappa^{*}\alpha^{2})]\;+\;j\omega LC \end{array}\right.$$

**Figure 3:**
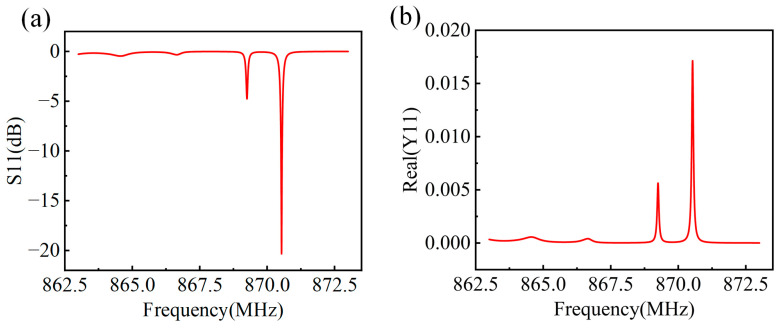
Simulated spectrum diagram of (**a**) S11 and (**b**) Y11 with a design frequency of 870 MHz based on COM model.

Among them, *N* is the number of electrodes of a single functional unit, and *L* is its length (L=Nλ). By calculating the P matrix of each functional unit (reflector, delay line, IDT), and finally cascading them to obtain a total matrix, the working performance of a designed device such as S11 and Y11 can be obtained. For example, the S11 and Y11 spectrums of a device with a design frequency of 870 MHz (50 IDT pairs, 120 reflector pairs, and 80λ aperture length) are shown in Figure 3a,b, respectively. Therefore, we can optimize the device performance by varying the IDT/reflector geometric parameters.

### 2.4. Optimization of IDT and Reflector

For a high-performance passive wireless sensing system, the designed SAW device should have a high Q factor and a good impedance match with the wireless interrogating system, which can be significantly influenced by the IDT geometric size parameters such as the number of IDT/reflector pairs and the aperture length. To quantitatively evaluate the performance of SAW device, it is essential to define a proper optimization criterion. Therefore, the Q factors of Z11, S11, and Y11 under varying IDT/reflector pairs and the aperture length have been systematically investigated to determine the optimization criterion. The Q factor value was calculated using the following equation:

(9) $$Q=\frac{f_{0}}{\Delta f}$$

where Δ*f* is the half-peak width of Z11, S11, and Y11 spectrums.

**Figure 4:**
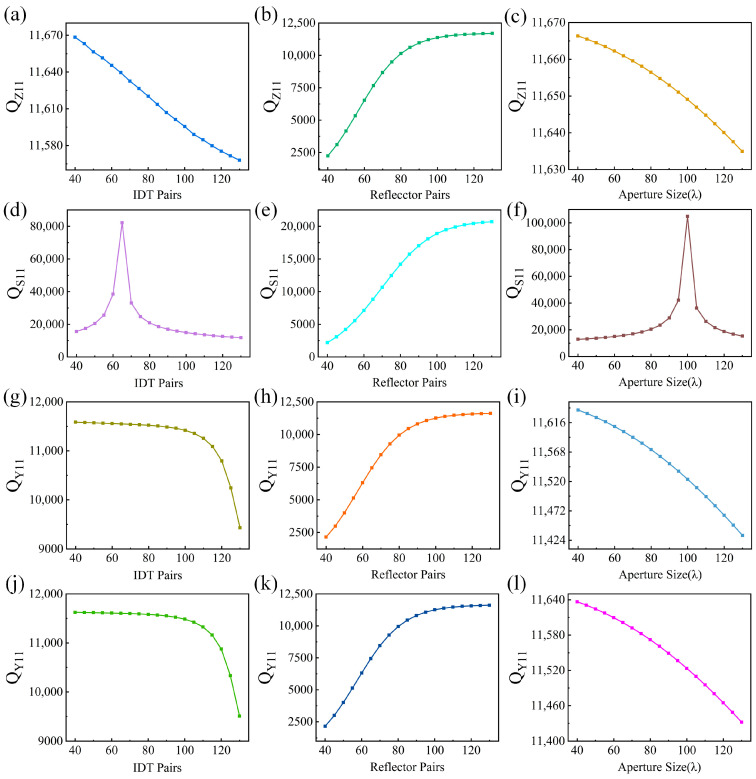
The variation trend of the Z11 Q factor under a different (**a**) IDT pair number, (**b**) reflector pair number, and (**c**) aperture size. The variation trend of the S11 Q factor under a different (**d**) IDT pair number, (**e**) reflector pair number, and (**f**) aperture size. The variation trend of the Y11 Q factor under a different (**g**) IDT pair number, (**h**) reflector pair number, and (**i**) aperture size. Considering the impedance matching performance, the variation trend of the Y11 Q factor under a different (**j**) IDT pair number, (**k**) reflector pair number, and (**l**) aperture size.

Take a SAW device with a design frequency of 870 MHz as an example. Firstly, the Q factor of Z11 has been calculated under varying IDT/reflector pairs and the aperture length. As shown in Figure 4a, it is clear that when the IDT pair numbers increase from 40 to 130, the Q factor slightly decreases from 11,670 to 11,560. As shown in Figure 4b, when the reflector pair numbers increase from 40 to 100, the Q factor gradually increases from 2230 to 11,300, and remains stable after that. For the aperture size increasing from 40*λ* to 130*λ* as shown in Figure 4c, the Q factor also slightly decreases from 11,666 to 11,634. It can be seen that the variation in IDT pairs and aperture size only lead to a 0.94% and 0.27% change in Z11 Q factor values, respectively, while increasing reflector pairs can significantly improve the Q factor of Z11 as much more acoustic energy can be reflected back with larger numbers of reflectors.

For the Q factor change in S11 as shown in Figure 4d–f, it is clear that the S11 Q factor changes significantly and an optimal value exists when the IDT pairs and aperture size changed, which is 65 and 100*λ* for IDT pairs and aperture size, respectively. As S11 usually represents the impedance matching performance between a SAW device and a 50 Ω port (e.g., antenna), the change in IDT pairs and aperture size leads to an impedance change in the SAW device. Therefore, a higher S11 Q factor means less energy loss between the SAW device and the antenna, which is preferred. For the change in reflector pairs, larger numbers of reflectors can also lead to a higher S11 Q factor.

As the spectrum of real (Y11) normally presents the intrinsic property of a SAW device, the real (Y11) Q factor changes under varying IDT/reflector pairs and the aperture length have also been summarized in Figure 4g–i. For the IDT pairs change as shown in Figure 4g, the Q factor keep stable when the number of IDT pairs increases from 40 to 100 pairs; after that, the Q factor declines dramatically. When increasing the number of reflector pairs, as shown in Figure 4h, the Q factor also gradually increases and becomes stable after about 100 pairs. For an increasing aperture size as shown in Figure 4i, the Q factor decreases in a nearly linear trend.

In summary, when considering the Q factor of real (Y11), the number of IDT pairs and aperture size should be as small as possible. However, considering the impedance matching performance (i.e., S11 Q factor), the number of IDT pairs and aperture size should not be too small. In practical, for a high-performance passive wireless sensing system based on SAW device, both the intrinsic energy loss in the SAW device and the energy loss between the SAW device and the antenna due to impedance mismatch should be minimized. Therefore, an optimization criterion considering both the Q factor of real (Y11) and S11 is determined. The optimization criterion first ensures the insertion loss of S11 is more than 15 dB. On this basis, the Q factor of real (Y11) is optimized to be as large as possible. Based on such an optimization criterion, the Q factor of real (Y11) has been calculated under varying IDT/reflector pairs and the aperture length as shown in Figure 4j–l. The variation trend is the same as that in Figure 4g–i, so we finally chose the Q factor of Y11 as the design criterion. It should be noted that the design results of considering (1) only the Y11 Q factor (i.e., Figure 4g–i) and (2) both the Y11 factor and S11 impedance match performance (i.e., Figure 4j–l) are exactly the same. It can be attributed to the narrow parameter sweep range set for IDT/reflector pair numbers and aperture length, and all the designs meet the criterion of an S11 more than 15 dB, which can also be seen in Figure 4d where even the minimum S11 Q factor exceeds 10,000.

**Figure 5:**
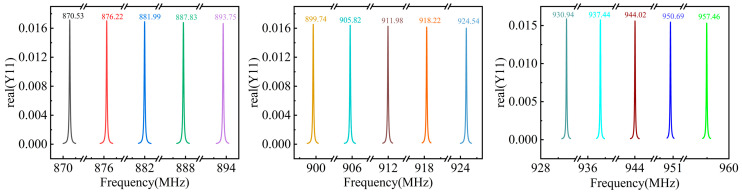
Spectrum of real (Y11) for the optimized SAW devices with 15 operating frequencies.

After the above comprehensive consideration, the IDT pairs, the reflector pairs, and the aperture size are set to be 50, 120, and 80λ, respectively. In addition, the maximum length and width of the designed SAW device are less than 1.035 mm and 0.3 mm, respectively. Moreover, using the above-mentioned optimization criterion, the designed real (Y11) values for each of the 15 frequencies are summarized in Figure 5.

## 3. Comparison of Experimental and Simulated Results

**Figure 6:**
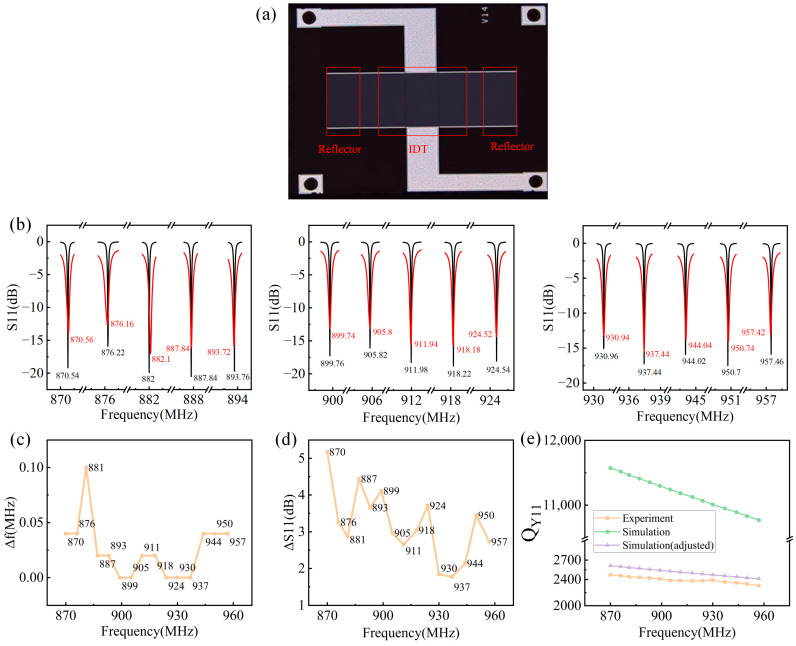
(**a**) A typical fabricated SAW device before packaging. (**b**) Comparison of simulated and experimental S11 results of SAW devices with 15 operating frequencies. (**c**) The resonant frequency differences of the simulated and experimental results. (**d**) The insertion loss differences of the simulated and experimental results. (**e**) The comparison of the experimental and simulated Y11 Q factor.

According to the above design results, the corresponding SAW devices have been fabricated. A typical fabricated SAW device is shown in Figure 6a before packaging, which is fabricated on YX-cut quartz. From Figure 6a, the patterns of IDT and reflectors can be clearly seen. The S11 spectra are measured using the network analyzer (Keysight E5071C). The experimental measured and simulated S11 spectra are shown in Figure 6b. The red and black curves present the experimental and simulated results, respectively, showing high consistency. The detailed frequency shifts for 15 operating frequencies are summarized in Figure 6c. The maximum frequency shift between the experimental and simulated results is 0.1 MHz. For the insertion loss shift as shown in Figure 6d, a maximum value of 5 dB occurred in SAW devices with a 870 MHz operating frequency. In addition, the comparison of the experimental and simulated Y11 Q factor is shown in Figure 6e, showing a high discrepancy in absolute value, but the trend is consistent. The discrepancy can be mainly attributed to the inaccurate propagation loss *γ* obtained from the literature review instead of the experimental measurement. Since a larger propagation loss leads to a smaller Q factor, a modification of propagation loss can be achieved based on experimental results for a more accurate COM model. The Q factor simulated based on the modified propagation loss is also shown in Figure 6d, and it is clear that the discrepancy is greatly decreased, and the COM model with modified propagation loss can be used for device design in the future.

## 4. Demonstration of Wearable Body Temperature Monitoring

**Figure 7:**
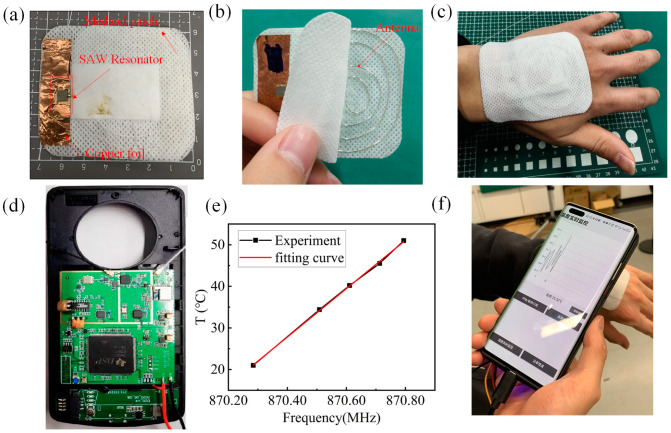
(**a**) The integrated medical patch for human body temperature monitoring, and its (**b**) buried antenna. (**c**) The medical patch can be placed on the hand for temperature monitoring. (**d**) The miniaturized interrogator integrated into a mobile phone case. (**e**) The temperature–frequency response of a ~870 MHz SAW temperature sensor. (**f**) Real-time display of temperature monitoring data on a mobile phone using the passive wireless sensing system.

Based on the above fabricated SAW device, a preliminary demonstration of wearable body temperature monitoring is conducted. A medical patch integrated with a SAW temperature sensor, coil antenna, and copper foil ground is shown in Figure 7a,b, which can be placed on the hand for temperature monitoring as shown in Figure 7c. When the antenna receives the interrogation signal from the previously developed miniaturized interrogator integrated into a mobile phone case as shown in Figure 7d, it will stimulate the SAW device to respond due to the piezoelectric effect. Based on the calibration results shown in Figure 7e, the interrogator can decouple the temperature-sensing information from the SAW resonant frequency signal, and finally display the real-time temperature monitoring data on the mobile phone as shown in Figure 7f. It is clear that the monitoring temperature is around 25 °C rather than 37 °C, which can be attributed to the following reasons: (1) The temperature of the human hand is usually lower than the body temperature and is affected by the indoor environment temperature [35]. (2) In the preliminary demonstration, the heat conduction between the human body and the SAW temperature sensor has not been optimized, and integrating flexible materials with high heat conductivity can solve this problem. Nevertheless, it is promising that the rationally designed ~915 MHz SAW device can be used in wearable applications.

## 5. Conclusions

In this paper, SAW devices with a resonant frequency distributed from 870 MHz to 960 MHz are proposed for wearable body temperature monitoring. The sensors are rationally designed based on FEM and COM models. The device parameters, including IDT pairs, aperture size, and reflector pairs, are systematically optimized, and the theoretical and experimental results showing high consistency. Finally, SAW temperature sensors with a quality factor greater than 2200 are obtained for real-time temperature monitoring ranging from 20 to 50 °C. Benefitting from the higher operating frequency, the size of the sensing system can be reduced for human body temperature monitoring, which may promote the development of SAW-based wireless passive temperature sensor and their application in intelligent medical treatment in the future.

## Data Availability

The original contributions presented in the study are included in the article, further inquiries can be directed to the corresponding authors.
